# First Contact: interprofessional education based on medical students' experiences from their nursing internship

**DOI:** 10.3205/zma001019

**Published:** 2016-04-29

**Authors:** Astrid Eich-Krohm, Alexandra Kaufmann, Kirstin Winkler-Stuck, Katrin Werwick, Anke Spura, Bernt-Peter Robra

**Affiliations:** 1 Otto-von- Guericke-Universität Magdeburg, Medizinische Fakultät, Institut für Sozialmedizin und Gesundheitsökonomie, Magdeburg, Germany; 2 Universitätsklinikum Magdeburg A. ö. R. Magdeburg, Germany; 3 Otto-von-Guericke-Universität Magdeburg, Medizinische Fakultät, Studiendekanat, Magdeburg, Germany

**Keywords:** Medical education, physicians-nurse relations, interprofessional relations, medical sociology, nursing internship

## Abstract

**Goal: **The aim of the course “interprofessional communication and nursing” is to reflect medical students’ experiences from the nursing internship. The content of the course focuses on barriers and support of interprofessional communication as a foundation for teamwork between nursing professionals and physicians. The nursing internship is for most medical students the first contact with nursing professionals and can lead to perceptions about the other group that might hinder interprofessional teamwork and consequently harm patients. To meet the demographic challenges ahead it is important to emphasize interprofessional education in the study of medicine and better prepare future physicians for interprofessional collaboration.

**Method: **The design of the course includes an assessment of a change in the students’ perceptions about nursing and interprofessional communication. The first class meeting presents the starting point of the assessment and visualizes students’ perceptions of nursing and medicine. The content of the following class meetings serve to enhance the students’ knowledge about nursing as a profession with its own theories, science and scholarship. In addition, all students have to write a research paper that entails to interview one nursing professional and one physician about their ideas of interprofessional communication and to compare the interviews with their own experiences from the nursing internship. To access what students learned during the course a reflective discussion takes place at the last meeting combined with an analysis of the students’ research papers.

**Results: **The assessment of the students’ perceptions about the nursing profession and the importance of successful interprofessional communication showed a new and deeper understanding of the topic. They were able to identify barriers and support measures of interprofessional communication and their own responsibilities as part of a team.

**Conclusion:** Interprofessional education is an important part of medical education and should be a topic from the beginning. The assessment of the course shows that it is possible and important to integrate the topic early in the curriculum.

## Zusammenfassung

**Ziel: **Das Ziel des Kurses „interprofessionelle Kommunikation und Pflege“ ist es, die Erfahrungen zu reflektieren, die Medizinstudierende während ihres Krankenpflegediensts gesammelt haben. Der Kurs befasst sich inhaltlich mit Barrieren sowie mit der Unterstützung interprofessioneller Kommunikation als Grundlage für die Zusammenarbeit zwischen Pflegepersonal und Ärzt_innen. Im Krankenpflegedienst treten die meisten Medizinstudierende zum ersten Mal mit dem Pflegepersonal in Kontakt. Dies kann zu bestimmten Einstellungen über die jeweils andere Gruppe führen, die eine interprofessionelle Zusammenarbeit behindern und somit Patienten schädigen können. Um auf die zu erwartenden demografischen Herausforderungen vorbereitet zu sein, ist es wichtig, die interprofessionelle Ausbildung im Medizinstudium zu verstärken, um zukünftige Ärzt_innen besser auf die interprofessionelle Zusammenarbeit vorzubereiten.

**Methode: **Die Kursplanung beinhaltet die Bewertung der veränderten Wahrnehmung der Studierende gegenüber der Pflege und der interprofessionellen Kommunikation. Das erste Seminar stellt dabei den Beginn der Bewertung dar. Es visualisiert, wie die Studierenden, Pflege und Medizin wahrnehmen. Die Inhalte der folgenden Seminare dienen der Vertiefung des Wissens der Studierenden über die Pflege als eigenständigen Beruf mit spezifischen Theorien und einem pflegewissenschaftlicher Forschungsbereich. Weiterhin müssen alle Studierenden eine Hausarbeit verfassen, für die sie ein Interview mit einem Angehörigen eines Pflegeberufes und einem Arzt führen sollen, bei dem deren interprofessionelle Kommunikation thematisiert wird. Diese Interviews sollen die Studierende mit ihren eigenen Erfahrungen aus dem Krankenpflegedienst vergleichen. Um herauszufinden, was die Studierenden während des Kurses gelernt haben, findet am letzten Termin eine reflektierende Diskussion statt, bei der auch eine Analyse der Hausarbeiten der Studierenden erfolgt.

**Ergebnisse: **Die Beurteilung dessen, wie die Studierenden Pflegeberufe wahrnehmen und die Wichtigkeit einer erfolgreichen interprofessionellen Kommunikation zeigte, dass ein neues und tieferes Verständnis für das Thema vorhanden war. Die Studierende konnten Hindernisse und unterstützende Maßnahmen für die interprofessionelle Kommunikation sowie ihre eigenen Verantwortlichkeiten als Teil eines Teams identifizieren.

**Fazit: **Die interprofessionelle Ausbildung ist ein wichtiger Bestandteil des Medizinstudiums und sollte von Beginn an thematisiert werden. Die Auswertung des Kurses zeigt, dass es möglich und wichtig ist, das Thema schon früh in den Studienplan zu integrieren.

## 1. Introduction

The goal of interprofessional education is preparing health professionals to work together in interprofessional teams for the good of the patient [1]. Demographic and economic developments call for an efficient and coordinated health care system that is able to act and react appropriately to these challenges. Nursing and medicine are professions that have to work together closely because nurses have the most contact with patients and physicians rely on nurses’ observations and reports to make the best choices regarding the effectiveness of medical therapy. This important link is regularly neglected in both educational pathways. Generally, nurses and physicians acquire interprofessional skills on the ward by watching how both groups interact with each other. Therefore, the “learning by doing approach” highly depends on real life role models both groups experience during their internships on the ward. 

In Germany most medical and nursing students are still educated apart from each other [2]. Whereas in many countries nursing has developed as a profession on equal footing with medicine based on a university education this is not the case in Germany [3]. Even though nursing has made a shift to become an independent profession with its own theories, science and scholarship, it is for the most part still an education that takes place in nursing schools in the secondary educational sector that are not equal to university studies of medicine in the tertiary sector [4]. A few medical schools and colleges of applied sciences for health professions have started to open their curricula and interconnect the students during their studies [1] but this is not enough to reach the majority of future nursing professionals and physicians. 

The medical school at the university medical center at Magdeburg has started to approach this issue by offering a structured nursing internship for medical students in their 1^st^ year by matching them with nursing students in their 2^nd^ and 3^rd^ years [5]. The course “interprofessional communication and nursing” [6] was developed as a part of the larger medical sociology curriculum [7] to emphasize communication, social research competency and research techniques. Legal requirements of nursing and medicine include explicitly the teaching of interprofessional skills as educational goals [http://www.gesetze-im-internet.de/_appro_2002/BJNR240500002.html], [8]. The objective of the course “interprofessional communication and nursing” is to give medical students a comprehensive idea of nursing as a foundation for their future work in interprofessional teams. The students’ experiences from their nursing internship will be used and expanded because most students have at least partially or completely finished the internship in their 1^st^ year. 

The nursing internship for medical students in Germany is part of the pre-medical education and a requirement to be eligible for the first state examination in medicine [http://www.gesetze-im-internet.de/_appro_2002/BJNR240500002.html]. The law states that medical students have to work for three months in a hospital or rehabilitation center to learn about the operation and organization of a hospital and the usual tasks of nursing [http://www.gesetze-im-internet.de/_appro_2002/BJNR240500002.html]. It is not meant to advice students in medical techniques and therapy. There is no individual evaluation required, at the end the student receives a written statement that he or she has fulfilled the requirements of the nursing internship handed out by the nursing director. Because there are no guidelines of what medical students should learn during the internship, the individual learning experience highly depends on the nursing professionals in the hospital. Discussions among medical students on web-blogs (for example: http://www.studis-online.de and http://www.medi-learn.de) show that for many the intent of the internship is unclear. This is critical because the nursing internship marks for many medical students the first contact with the nursing profession in a hospital. To evaluate how the course structure and content helps medical students to reflect the topic of interprofessional teamwork and change their perceptions about nursing a qualitative assessment was developed. The following article describes the course concept and its results.

## 2. Description of the course “interprofessional communication and nursing”

The aim of the course is to reflect and evaluate medical students’ experiences from the nursing internship. The focus is on barriers and support of interprofessional communication as a foundation for teamwork between nurses and physicians. The course is part of the medical sociology curriculum and offered at this point to medical students only. It entails 14 credit hours during the 2^nd^ semester. It is co-taught by a nursing professional with advanced training from the intensive care unit and a sociologist/nursing educator with many years of experience. By the time the course takes place the medical students have finished their nursing internships at least partially. This was the second time the course was taught and from the experiences a year before the assessment of the learning was built into the course structure. At the same time this is also one of the limitations of this assessment because the course was taught this way for the first time and with 18 participating students. 

**Figure 1:**
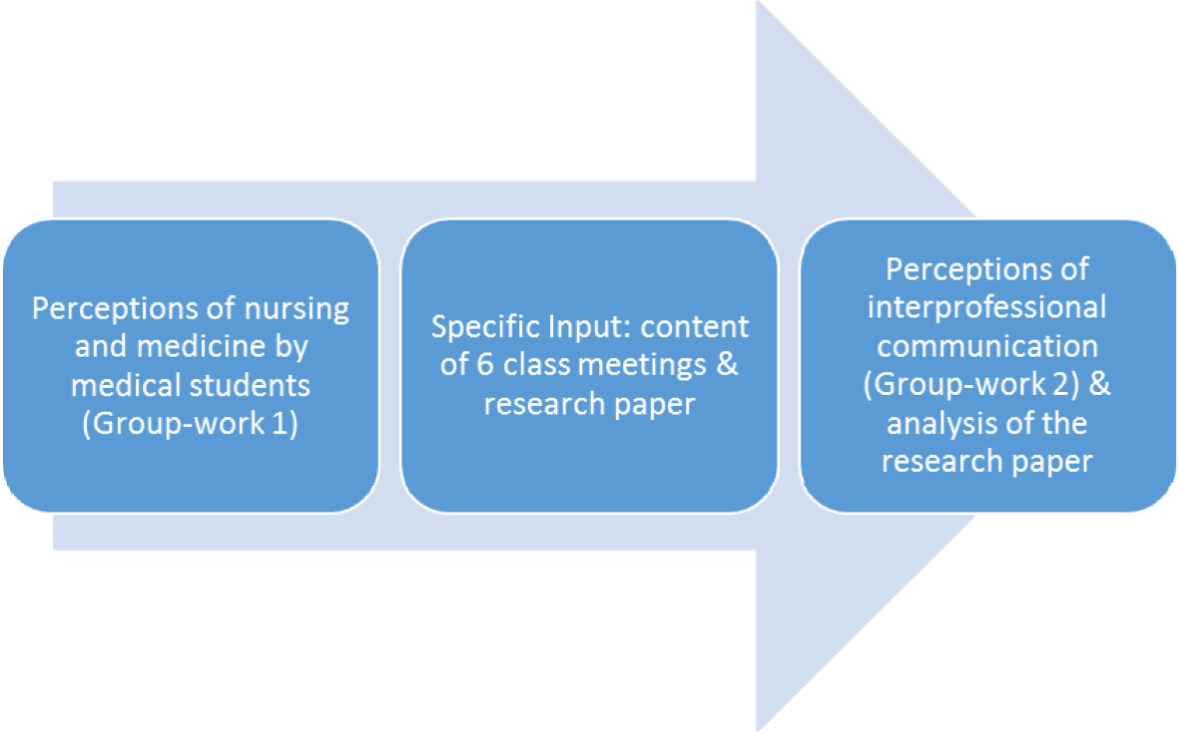
Course structure of interprofessional communication and nursing

The assessment of the students’ learning in the course entails a comparison of their perceptions about the topic when they enter the course to their perceptions at the end of the course connected with an analysis of their research paper. The first class set the stage for the following meetings. Group-work 1 presents the starting point of the students’ journey in the course and visualizes their perceptions of nursing and medicine. The content of the class meetings and the research paper serve to enhance the knowledge about nursing as a profession and barriers/support of interprofessional communication. To evaluate what students learned during the course a reflective discussion in groups took place at the last meeting. The results of group-work 2 were then compared with the individual research papers that students had to hand in a week after the course concluded (see figure 1 (Fig. 1)).

Based on the results of group-work 1 the content of the following six class meetings focused on nursing as a profession with its own techniques (see table 1 (Tab. 1)), [9], [10]. 

Subsequently, the 2^nd^ class meeting took place in the skills-lab of the university hospital. The goal was to evaluate the knowledge of the medical students regarding nursing by discussing specific techniques hands-on. The students knew some of the techniques that nursing professionals use to prevent complications due to hospital stays, such as venous thromboembolism prophylaxis, but they did not know the concepts behind. During the session in the skills-lab the students learned that nursing care depends on the individual patient and the situation in which it happens. It is complex and requires communicative, observational and emotional skills, empathy and intuition for patients’ individual situation to understand their needs in that moment of care [11]. To emphasize this point, a student was asked to take on the patient role and then to explain how she felt while being moved in bed by two classmates. Her response underlined the statement above, she was afraid to fall out of bed because her classmates didn’t explain to her what they would do next. She felt “invisible” because they didn’t talk with her instead they talked with each other. The technique hurt because the classmates didn’t work together. Overall, the student experienced the whole situation as very uncomfortable. To support the theoretical content of the class meetings the accompanying literature for the course came from nursing sources only. 

The research paper required the students to interview a nurse and a physician about their experiences with interprofessional communication. The interviews were guided by the question: what makes interprofessional communication between nursing professionals and physicians successful? The students asked interview participants to think of a colleague from the other profession with whom they like to work together and explain why. If participants couldn’t think of a colleague they should explain their understanding of interprofessional communication. The length of the research paper was five to ten pages and should link the course literature with statements of the interview participants and the students’ own experiences from the nursing internship. A week after the course ended all students had to hand in the research paper. The analysis of the research paper focused only on the conclusion the students wrote. The intent was to evaluate what the students learned from the interviews and not the original interview content. Eighteen students participated and wrote on average seven pages. The conclusion should answer the following questions: what did you learn from the interviews, what surprised you the most and what do you take away from it for your future work as a physician? The conclusions of the papers varied from one-and-a-half to three pages. All conclusions were transferred into one document and analyzed qualitatively in finding common themes in the students’ conclusions [12]. Overall, the conclusions explained in more detail the issues that students acknowledged in group-work 2 at the end of the course.

## 3. Results: perceptions of nursing and medicine

To be able to assess what students’ learned in the course they were first asked about their ideas of nursing and medicine (group-work 1). Medical students do not enter the internship as blank slates. Instead they start with their individual ideas about nursing that have been partly created by society and by their individual nursing internship experience. The results of group-work 1 (see table 2 (Tab. 2)) show that nursing is still perceived as a physician’s assistance job and not as a profession in its own right. 

The hierarchical order between nursing and medicine is well established in group-work 1. The students used educational length, salary differences, type of work and more (physicians) or less (nurses) freedom of decision making as markers of professional hierarchy. According to the students, the most important part of nursing is to help the physician in his daily quest. This perception rather hinders than helps interprofessional teamwork. 

Table 3 (Tab. 3) from the end of the course illustrates the change in students’ perceptions about the nursing profession compared to table 2 (Tab. 2) from the beginning of the course. The students are able to identify challenges of and solutions for successful interprofessional communication more profound than in table 2 (Tab. 2) because their understanding of nursing has changed. Whereas in table 2 (Tab. 2) nursing was only perceived as a supporting profession for physicians they are now acknowledging nursing as a profession in its own right. For example, the students view hierarchical differences as a challenge and conclude that both professions can learn from each other. They attest both groups to have experience and professional knowledge and that joint activities, such as medical rounds in which nursing staff and physicians participate are one solution to foster interprofessional teamwork. 

The change in students’ perceptions is probably not only based on the content of the class meetings but also influenced by the interviews the students had to conduct for their research paper. The analysis showed that themes acknowledged in group-work 2 are also found in the individual papers. Further analysis of the students’ research demonstrates that they describe problems and solutions based on the interviews and the literature. The themes that students most often cited as barriers of interprofessional communication are: neglect of social norms and values in daily communications, hierarchical differences between nursing and medical professions, academic versa apprenticeship nursing education, and stress at work due to understaffing as organizational limitation. Factors of successful interprofessional communication are all actions that support a collaborative work environment, for example joint medical rounds and when both groups participate in periodical meetings, such as shift change. 

## 4. Discussion

The students’ perceptions of nursing as a physician’s assistance job reflect common societal views [13]. However, an important requirement for successful continuation of care for patients between health professions depends on excellent primary care nurses who can make independent decisions [13]. Many countries have reacted to this issue by establishing academic programs of nursing but Germany still struggles with this issue. The assumption is that support of activities of living [14], emotional and social care work, as well as monitoring patients is not science instead it is perceived as skilled work in comparison to medicine. This is based on a definition of nursing that is divided into basic and specialist nursing care. Basic care is support of the activities of living and specialist care is therapeutic medical care that partially overlaps with physician’s work, for example giving an injection [11]. The complexity of nursing is lost by this distinction [11], which mirrors the historical development of nursing as a female profession based on women’s roles as caretakers of family members and as serving members of religious groups [15]. The research paper allowed the students to discuss these issues with representatives of both professions and at the same time reflect their own experiences from their nursing internship. Not surprisingly, the themes of the research papers and the issues presented in table 3 (Tab. 3) are matching because the students had finished the interviews shortly before the last group-work.

### 4.1. Neglect of social values and norms as barriers of interprofessional communication

The most important issue for the students was how much social values and norms were ignored by nursing professionals and physicians in the treatment of each other. The students agreed that respect, tolerance, trust, honesty and openness should guide how both groups engage with each other. As one student put it: 

“I was surprised by the fact that in the day-to-day business of a hospital ward common social values don’t seem to have a meaning anymore. Average communications are difficult matters. It seems that two completely different worlds are colliding even though they have the same goal namely to work in the best interest of the patient.” 

As mentioned before, nursing and medical students are not educated together. They are socialized into their prospective professional role by members of their own group. Therefore traditional views about the other group will be socially reproduced making it difficult for subsequent generations of nurses and physicians to overcome those perceptions and to reach a “we” instead of “they” in their joint quest of helping patients. 

#### 4.2. Hierarchical Differences and issues of power

The students acknowledged that hierarchical differences are often in the way of teamwork in vertical hierarchies as one student describes:

“One point in both interviews was the strict hierarchy in the hospital. Nurses treat physicians with too much respect. I don’t think that we can abolish the hierarchy because the physician is ultimately the person who has the final word. But I do agree with my interview participants that the groups should meet each other on equal footing.” 

The term “to meet each other on equal footing” is a relatively new idea to describe the nursing – physician relationship in Germany. The work between both groups naturally overlaps and a number of medical tasks, such as drawing blood from a patient can be done by a nurse, a medical student or a physician. However, in the German context the physician is authorized to issue directives and give orders to nursing staff. The final responsibility for medical orders lies with the physician [16]. This situation has historical roots in the development of both professions, one as a three-year education on the secondary and the other as a university program on the tertiary level. Only recent changes in nursing law [8] have defined specific tasks, techniques and processes based on nursing scholarship as independent nursing work. Some might view these developments as dangerous because they define for the first time that nursing is based on nursing science and, therefore, in parts independent of medicine. This issue also reflects power and authority between both groups. In Group-work 2 students identified encounters of older nurses and young physicians as problematic. The older nurse has accumulated substantial practical experience giving her some power over younger physicians despite her “lower ranking” in the overall hierarchy. Physicians at the beginning of their careers do not have the practical experience but accumulated theoretical knowledge in years of study that they like to use to show their power. The students came to the conclusion that it’s best if both groups can learn from each other to find the best treatment for the patient. 

#### 4.3. Academic development of the nursing profession

The ever expanding medical knowledge calls for academically trained nurses who can understand complex medical treatments. However, there is still a discussion among health professionals and politicians [17] if Germany is in need of nurses with a B.A. One student described the reactions she received when she asked her interview participants about academic nursing:

“The physician spoke very clearly against an academic program of nursing. In his view a study of nursing won’t change communication and teamwork between nurses and physicians. The nurse on the other hand, supported the idea of an academic nursing program and views it as a chance for nursing professionals to be taken seriously.” 

A higher number of academically trained nursing professionals will eventually lead to better teamwork with physicians which can be already observed in intensive care units today [18]. Academic nursing programs can help to overcome role conflicts with physicians based on unclear responsibilities. Of course, it might also mean for physicians to share power. All programs that educate health professionals (regardless if academic or not) should integrate interprofessional education to foster a more cooperative environment among them [19]. 

#### 4.4. Organizational barriers of interprofessional communication 

Many of the students believe that stress on the ward is a factor causing interprofessional communication to go in the wrong direction. Nursing professionals experience stress through irregular shift work, when legal breaks during work cannot be taken, unplanned overtime and a low salary [20]. These are structural reasons caused by a shortage of nursing professionals. Of course, students cannot change those larger issues and they realize that. However, they do agree that they can at least as individuals adhere to common social values. One student writes: 

“I take with me for my future career that I am responsible for my treatment of others. I want to stay sensible about this topic and to be polite, friendly and respectful towards everyone else. After my nursing internship I decided never to forget the hard physical work that nurses have to do and not take it for granted. I think many physicians forget about it even though they all went through the nursing internship.”

The students reflect correctly that large organizations influence how people work together and the negative impact this can have. The stress is on both ends because physicians also experience overtime and too much work [18]. Economic and efficiency goals set by health organizations, such as hospitals have limited physicians’ freedom of decision making [21]. 

#### 4.4. Examples for change

One of the requirements of the writing assignment was to ask for positive examples of interprofessional communication. Besides good social values students described that joint activities on the ward can support teamwork for example, when members of both professions join medical rounds and physicians are present when the nursing shift changes. In both situations questions regarding specific patients and treatments can be clarified and problems discussed. Nevertheless, nursing staff and physicians have to be prepared for joint rounds to make it a success and health organizations should provide interprofessional training for those who already finished their education [20]. Another issue is the formality on hospital wards. Young nurses and physicians refer to each other more informal by using the first name of the person and “you.” Whereas older nursing professionals prefer the more formal last name to address and be addressed by others. It means that older nursing professionals adhere to social norms that emphasize the hierarchical order whereas younger professionals on both sides are able to interact without it. A rethinking of the hierarchical order seems to have started among the next generation of health professionals. 

## 5. Conclusions

The assessment of the course interprofessional communication and nursing shows how important it is to raise medical students’ awareness about this topic. The nursing internship is the first contact between medical students and the nursing profession and should be reflected more deeply. The results show that students are able to acknowledge the views of both professions and realize how important socially accepted values as a basis for good communication are. They conclude that learning from each other supports interprofessional communication better than emphasizing hierarchies. At the last class meeting the co-teachers asked the students if the course had been helpful to them. The response was a clear ‘yes’ but with reservations. The students who already finished their nursing internship explained that they wished to have had this knowledge before they started. The students who still had weeks of the internship left were glad to be able to try some things out. 

Medical sociology can help to incorporate interprofessional education because of its early appearance in the medical curriculum. Social values and norms, power and organizational structures, social roles, social class, and gender roles are important concepts that make a distinct contribution to both professions. More importantly, it can bridge the gap between theory and practice. Stereotypes about other health professions can manifest and lead to mistakes that can endanger patients’ health and lives [22]. Hence, it is important for successful interprofessional teamwork to not only include it in educational guidelines but to make it a part throughout all phases of nursing and medical programs. International comparison shows that Germany lags behind other countries in interprofessional teamwork [1], [23]. Nursing and medicine are the two professions that carry highest responsibility for the well-being of patients. To understand, accept and work with each other these professions have to be educated at least in parts together. Medical programs and nursing schools cannot wait to meet these challenges anymore. The structured internship as well as the course interprofessional communication and nursing are steps in the right direction but as of now they are not offered to all medical and nursing students. The aim is to develop a course that will be taken together by nursing and medical students and to make it a part of the core curriculum. 

## Competing interests

The authors declare that they have no competing interests.

## Figures and Tables

**Table 1:**
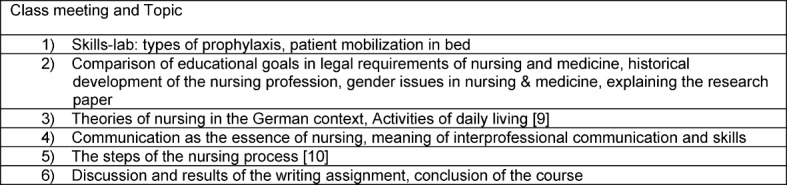
Overview of topics discussed in 6 class meetings

**Table 2:**
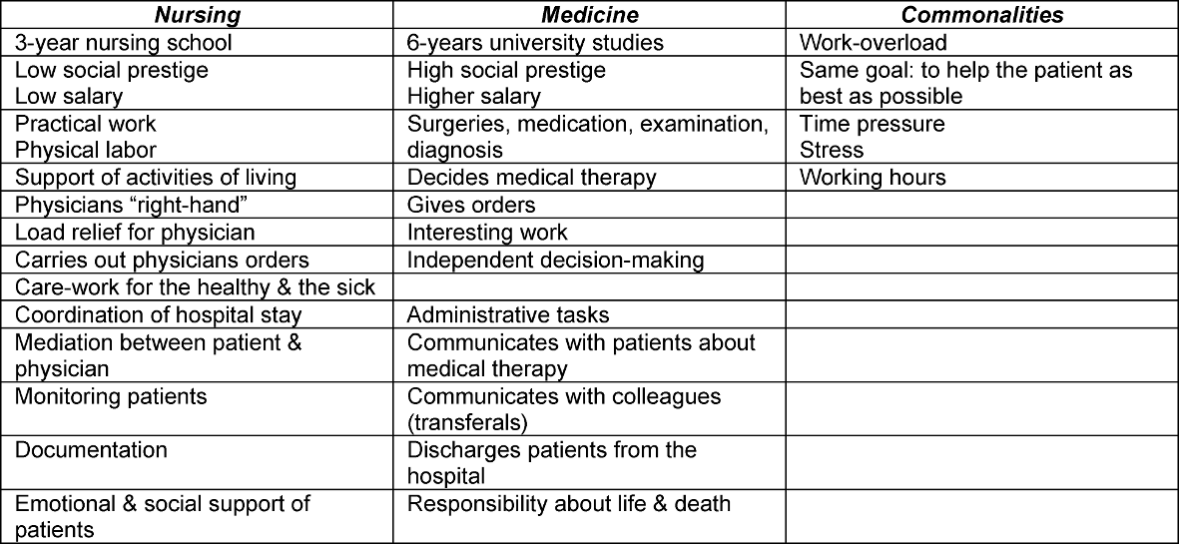
Medical students’ perceptions of nursing and medicine (start of course)

**Table 3:**
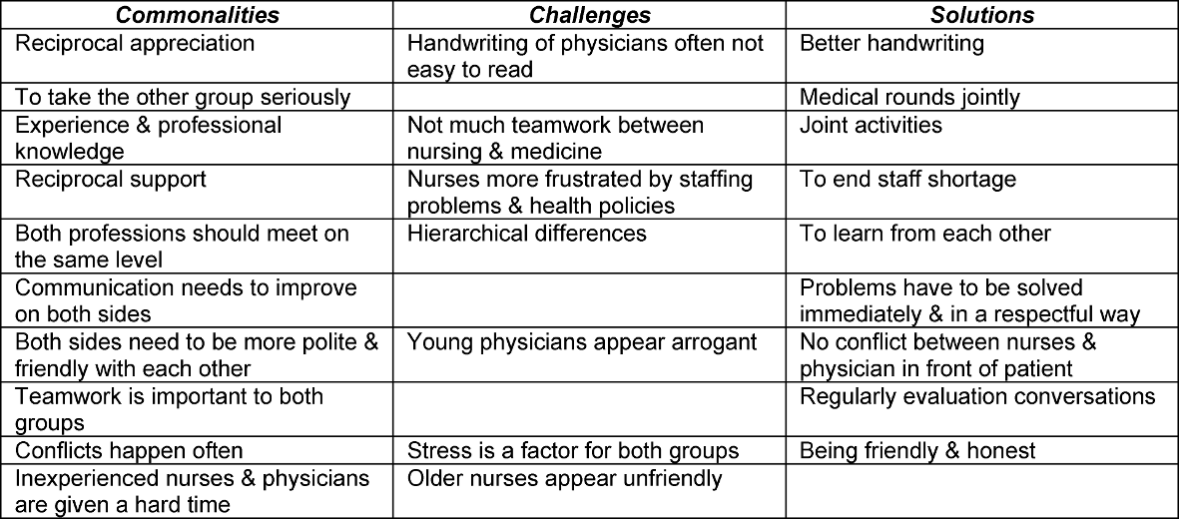
Medical students’ perceptions of interprofessional communication between nursing professionals and physicians (end of course)
